# Vitamin D supplement use in Egypt and Jordan and associated factors: A survey-based study

**DOI:** 10.1371/journal.pone.0357029

**Published:** 2026-08-28

**Authors:** TaqiAldeen Khabour, Omar F. Khabour, Salwa F. M. Hassanein

**Affiliations:** 1 Department of Internal Medicine, Faculty of Medicine, Jordan University of Science and Technology, Irbid, Jordan; 2 Department of Medical Laboratory Science, Faculty of Applied Medical Laboratory, Jordan University of Science and Technology, Irbid, Jordan; 3 Department of Food Science and Technology, Ain Shams University, Cairo, Egypt; Universitas Muhammadiyah Aceh, INDONESIA

## Abstract

The use of vitamin supplements is widespread throughout the world. In the Middle East, vitamin D deficiency is common. Therefore, this study aimed to examine the prevalence of vitamin D supplement use and associated factors among Egyptians and Jordanians. In addition, practices for maintaining adequate vitamin D levels were investigated. The study was cross-sectional and included 1,181 subjects from Jordan and Egypt. Participant recruitment was conducted from April to October 2024. The results showed that approximately one-third of the sample (36.7%) were using vitamin D supplements. Vitamin D supplementation was more prevalent among Jordanians (45.2%) than Egyptians (27.2%, P < 0.01). Factors influencing vitamin D supplement use included female gender, high income, chronic disease, laboratory testing for vitamin D, and awareness of deficiency symptoms. Among the practices participants used to maintain adequate vitamin D levels were sun exposure (84.6%), consuming vitamin D-rich foods (79.6%), regularly monitoring the blood vitamin D levels (49.5%), and using vitamin D supplements (36.7%). The internet, university/school curricula, and healthcare providers were the main sources of information related to vitamin D. In conclusion, vitamin D supplementation is common among study participants and is associated with several factors. The study findings can inform interventions aimed at managing vitamin D deficiency/insufficiency in the region.

## Introduction

The use of multivitamin supplements is widespread throughout the world [1,2]. According to the dietary supplement industry, the purpose of multivitamin supplements is to enable people to meet the body’s needs for vitamins that are not adequately absorbed through food intake or in individuals who do not regularly follow a balanced diet [3,4]. Vitamin supplements are also recommended for people with vitamin deficiencies related to health conditions such as malnutrition, infections, pregnancy, and others [5,6]. Underscoring this need, a study on an urban Mediterranean population revealed a high prevalence of hypovitaminosis D in both mothers and neonates [7].

In the Middle East and North Africa (MENA) region, several studies have shown high rates of vitamin D deficiency/ insufficiency [8,9]. For example, vitamin D levels were found to be deficient/insufficient in approximately two-third of children and the majority of adults (85%) in Jordan [10,11]. A similar picture has reported in Egypt, where most adults and about 70% of children suffer from a deficiency or insufficiency of vitamin D [12,13]. High rates of vitamin D deficiency/insufficiency have been reported in several countries in the MENA, including Saudi Arabia, the United Arab Emirates, Lebanon, Iraq, and Tunisia [14–18]. Vitamin D deficiency is a serious condition associated with health issues such as bone diseases, cardiovascular diseases, autoimmune conditions, and others [9,19].

Managing vitamin D deficiency/insufficiency involves using high doses of vitamin D supplements to reach optimal levels, followed by low maintenance doses [20,21]. In addition, to maintain adequate vitamin D levels, sun exposure and a diet rich in vitamin D, such as oily fish, egg yolks, and fortified foods, are recommended [22]. Furthermore, raising awareness about the prevention and management of vitamin D deficiency/insufficiency is crucial, especially given its prevalence in the MENA region [23,24]. Therefore, the current study focused on vitamin D supplementation use and related factors among Egyptians and Jordanians. The study also examined practices for maintaining adequate vitamin D levels among the study participants.

## Methods

### Study design

The study was cross-sectional, self-report, and questionnaire-based in its design. The recruitment process took place between 15/04/2024 and the 30/10/ 2024. The inclusion criteria were that the individual be an adult (over 18 years old) and a resident of Egypt or Jordan. Participants were recruited from public places and via social media platforms using a convenient sampling method.

The “G-power” program was used to compute the sample size assuming a moderate effect size, an alpha coefficient of 0.05, and a 1-beta coefficient of 0.95. The selected sample (n = 1,181 participants) slightly exceeded the required sample (n = 1,073 participants).

### Ethical issues

Participants gave their written informed consent online before completing the questionnaire. The study was approved by the “Institutional Review Board of Jordan University of Science and Technology” (approval code: 2024/168/7). The questionnaire was anonymous (no personal or identifying information was collected). Only adult participants were allowed to participate and children were excluded from the study.

### The study instrument

The study instrument was prepared using “Google Forms” and covered several questions to achieve the study objectives. The questionnaire’s questions were preceded by an introductory page that addressed aspects such as the title of the study, objectives and significance, voluntary participation, instructions, and an online consent. Demographic data for participants (age, sex, nationality, highest degree obtained, job status, and health insurance coverage) were collected in the first section. Data related to health status, vitamin D supplementation, and laboratory vitamin D level test were collected in the second section. Finally, data on the practices followed by participants to maintain adequate blood levels of vitamin D and sources of information related to vitamin D were collected in the last section. Two experts reviewed the instrument to ensure its suitability to the content and the clarity of its questions. A pilot test was conducted in both Egypt and Jordan to validate the study instrument. The questions were then revised based on feedback.

### Statistical analysis

SPSS software was used for all statistical analysis. Multivariate binary logistic regression was used to determine factors predicting vitamin D supplementation in the whole sample and in each country individually. Descriptive statistics were used to analyze practices and knowledge sources related to vitamin D. A P < 0.05 indicates statistical significance.

## Results

The study sample includes 1181 participants from Egypt (47.6%) and Jordan (52.4%, Table 1). The majority of participants were young adults (62.4%, < 40 years old), employed in the public and private sectors (57.1%), residing in cities (65.6), holding a university degree (68.2), being middle-income (68.2), and having health insurance (78.7%). Female participants represented 53.5% of the sample. About a quarter of the sample (27.7) suffered from a chronic disease (Table 1). Approximately 36.7% of the sample were using vitamin D supplements, including 45.2% Jordanians and 27.2% Egyptians (P < 0.01).

**Table 1:** Demographics of the study participants.

Category	Subcategory	Egyptian	Jordanian	Total
Age	18-30	234(47.3)	261(52.7)	495(41.9)
	31-40	126(52.1)	116(47.9)	242(20.5)
	41-50	100(46.1)	117(53.9)	217(18.4)
	51-60	56(42.1)	77(57.9)	133(11.3)
	>60	46(48.9)	48 (51.1)	94(8.0)
Sex	Male	272(49.5)	277(50.5)	549(46.5)
	Female	290(45.9)	342(54.1)	632(53.5)
Nationality	Egyptian	562(47.6)	–	562(47.6)
	Jordanian	–	619(52.4)	619(52.4)
Job status	Governmental sector	211(37.5)	193(31.1)	404(34.2
	Private Sector	130(23.1)	141(22.8)	271(22.9)
	Retired	33(5.9)	29(4.9)	62(5.2)
	Not employed	188(33.5)	256(41.2)	444(37.6)
Place of living	City	342(60.8)	433(70.0)	775(65.6)
	Village	220(39.2)	186(30.0)	406(34.4)
Educational level	≤ High school	48(8.5)	50(8.1)	98(8.3)
	Diploma	55(9.8)	30(4.8)	85(7.2)
	University degree	388(69.0)	418(67.5)	806(68.2)
	High Education	71(12.6)	121(19.6)	192(16.3)
Reported income status	High income	116(20.6)	103(16.7)	219(18.5)
	Medium income	365(64.9)	441(71.2)	806(68.2)
	Low income	81(14.5)	75(12.1)	156(13.2)
Health insurance	Governmental insurance	301(53.6)	386(62.4)	687(58.2)
	Private insurance	110(19.6)	132(21.3)	242(20.5)
	No insurance	151(26.9)	101(16.3)	252(21.3)
Presence of chronic diseases	Yes	212(37.7)	115(18.6)	327(27.7)
	No	350(62.3)	504(81.4)	854(72.3)

Table 2 shows the multivariate binary logistic regression analysis of factors associated with vitamin D use in the entire sample. Vitamin D use was associated with female gender (P < 0.001), higher income (P < 0.05), having a chronic disease (P < 0.001), laboratory testing for vitamin D (P < 0.001), and awareness of vitamin D deficiency symptoms (P < 0.01). There was no association between any of the other factors and vitamin D use in the entire sample.

**Table 2:** Multivariate regression analysis of demographic variables associated with vitamin D use among the whole sample.

		B (S.E.)	Wald	df	Sig.	Exp(B) (95% CI)
Age	>60		3.361	4	0.499	
	18-30	−0.616 (0.34)	3.121	1	0.077	0.540 (0.273-1.07)
	31-40	−0.445 (0.33)	1.797	1	0.180	0.641 (0.335-1.228)
	41-50	−0.317 (0.32)	0.930	1	0.335	0.728 (0.383-1.387)
	51-60	−0.307 (0.33)	0.866	1	0.352	0.736 (0.386-1.404)
Sex	Female	0.841 (0.17)	22.80	1	**0.000**	2.319 (1.642-3.275)
Nationality	Egyptian	−0.109 (0.17)	0.393	1	0.531	0.897 (0.638-1.260)
Job status	Gov. sector		1.28	3	0.732	
	Not employed	−0.222 (0.25)	0.774	1	0.379	0.801 (0.489-1.313)
	Priv. Sector	−0.124 (0.22)	0.320	1	0.572	0.883 (0.575-1.358)
	Retired	0.174 (0.36)	0.226	1	0.635	1.19 (0.581-2.437)
Place of living	Village	−0.064 (0.16)	0.145	1	0.703	0.938 (0.676-1.302)
Educational level	University		2.270	3	0.518	
	Diploma	−0.147 (0.31)	0.223	1	0.637	0.863 (0.468-1.591)
	MSc/PhD	−0.309 (0.21)	2.124	1	0.145	0.734 (0.485-1.112)
	School	0.018 (0.30)	0.004	1	0.952	1.018 (0.562-1.844)
Reported income status	Medium		8.691	2	**0.013**	
	High	0.470 (0.20)	5.102	1	**0.024**	1.601 (1.064-2.408)
	Low	−0.395 (0.24)	2.613	1	0.106	0.674 (0.418-1.087)
Health insurance	Priv. insurance		0.892	2	0.640	
	Gov. insurance	0.044 (0.20)	0.047	1	0.829	1.045 (0.700-1.561)
	No insurance	−0.151 (0.24)	.373	1	0.541	0.860 (0.530-1.395)
Chronic diseases	Yes	0.826 (0.18)	19.354	1	**0.000**	2.284 (1.581-3.299)
Performed test	Yes	2.238 (0.17)	164.59	1	**0.000**	9.377 (6.661-13.20)
Knowing Symptoms	Yes	0.519 (0.18)	7.923	1	**0.005**	1.680 (1.171-2.410)
	Constant	1.456 (0.45)	10.460	1	0.001	4.287

Subsequently, regression analysis was conducted for each country individually. Among Egyptians (Table 3), age and income were found to be associated with vitamin D use. Compared to the over-60 age group, those under 50 were less likely to use vitamin D supplements (P = 0.013–0.057). Compared to the middle-income group, higher income group was associated with increased vitamin D supplement use, while lower income group was associated with decreased vitamin D supplement use (P < 0.05). In addition, female gender (P < 0.05), having a chronic disease (P < 0.05) and having a vitamin D blood level test (P < 0.001) were associated with vitamin D supplement use (Table 3).

**Table 3:** Multivariate regression analysis of demographic variables associated with vitamin D use among Egyptian.

Variable	Category	B (S.E.)	Wald	df	Sig.	Exp(B) (95% CI)
Age	>60		7.006	4	0.136	
	18-30	−1.283 (0.55)	5.448	1	**0.020**	0.28 (0.094-0.814)
	31-40	−1.017 (0.53)	3.615	1	0.057	0.36 (0.127-1.032)
	41-50	−1.368 (0.55)	6.157	1	**0.013**	0.25 (0.086-0.750)
	51-60	−0.757 (0.52)	2.075	1	0.150	0.47 (0.167-1.314)
Sex	Female	0.766 (0.30)	6.464	1	**0.011**	2.15 (1.192-3.885)
Job status	Gov. sector		2.862	3	0.413	
	Not employed	−0.163 (0.42)	0.149	1	0.699	0.85 (0.371-1.943)
	Priv. Sector	−0.075 (0.35)	0.044	1	0.833	0.928 (0.463-1.86)
	Retired	−0.976 (0.58)	2.793	1	0.095	0.377 (0.120-1.184)
Place of living	Village	0.299 (0.27)	1.206	1	0.272	1.34 (0.791-2.297)
Educational level	University		1.604	3	0.659	
	Diploma	0.318 (0.43)	0.532	1	0.466	1.37 (0.585-3.230)
	MSc/PhD	0.350 (0.33)	1.067	1	0.302	1.42 (0.731-2.755)
	School	−0.073 (0.47)	0.024	1	0.878	0.929 (0.366-2.361)
Reported income status	Medium		10.200	2	**0.006**	
	High	0.644 (0.31)	4.204	1	**0.040**	1.91 (1.029-3.527)
	Low	−0.878 (0.41)	4.545	1	**0.033**	0.42 (0.186-0.932)
Health insurance	Priv. insurance		0.344	2	0.842	
	Gov. insurance	0.126 (0.33)	0.145	1	0.703	1.13 (0.594-2.165)
	No insurance	−.049 (0.40)	0.015	1	0.904	0.95 (0.432-2.102)
Chronic diseases	Yes	0.669 (0.26)	6.304	1	**0.012**	1.95 (1.158-3.293)
Performed test	Yes	2.52 (0.24)	102.86	1	**0.000**	12.46 (7.657-20.30)
Knowing Symptoms	Yes	0.40 (0.27)	2.145	1	0.143	1.50 (0.872-2.580)
	Constant	0.536 (0.68)	0.615	1	0.433	1.710

Among Jordanians (Table 4), female gender, retirement, having a chronic disease, having a vitamin D blood level test, and awareness of vitamin D deficiency symptoms were all associated with higher vitamin D supplement use (Table 4, P < 0.05). On the other hand, obtaining a higher degree (PhD/master’s) was associated with lower vitamin D use (P < 0.05).

**Table 4:** Multivariate regression analysis of demographic variables associated with vitamin D use among Jordanians.

Variable	Category	B (S.E.)	Wald	df	Sig.	Exp(B) (95% CI)
Age	>60		4.742	4	0.315	
	18-30	−0.646 (0.47)	1.814	1	0.178	0.52 (0.205-1.342)
	31-40	−0.420 (0.45)	0.870	1	0.351	0.65 (0.272-1.588)
	41-50	0.057 (0.44)	0.017	1	0.897	1.05 (0.446-2.511)
	51-60	−0.324 (0.45)	0.509	1	0.475	0.72 (0.297-1.761)
Sex	Female	0.958 (0.23)	17.228	1	**0.000**	2.61 (1.658-4.099)
Job status	Gov. sector		4.570	3	0.206	
	Not employed	−0.086(0.33)	0.067	1	0.796	0.918 (0.481-1.754)
	Priv. Sector	−0.083 (0.29)	0.079	1	0.779	0.920 (0.515-1.644)
	Retired	1.154 (0.59)	3.833	1	**0.050**	3.172 (0.999-10.07)
Place of living	Village	−0.310 (0.23)	1.736	1	0.188	0.73 (0.462-1.163)
Educational level	University		7.396	3	0.060	
	Diploma	−0.682 (0.47)	2.109	1	0.146	0.51 (0.201-1.269)
	MSc/PhD	−0.724 (0.29)	6.004	1	**0.014**	0.49 (0.272-0.865)
	School	−0.316 (0.43)	0.537	1	0.464	0.73 (0.313-1.698)
Reported income status	Medium		4.154	2	0.125	
	High	0.581 (0.30)	3.587	1	0.058	1.79 (0.980-3.262)
	Low	−0.223 (0.32)	0.460	1	0.498	0.80 (0.419-1.525)
Health insurance	Priv. insurance		0.383	2	0.826	
	Gov. insurance	−0.039 (0.27)	0.021	1	0.886	0.96 (0.562-1.644)
	No insurance	−0.195 (0.33)	0.332	1	0.565	0.82 (0.423-1.598)
Chronic diseases	Yes	1.017 (0.29)	12.179	1	**0.000**	2.76 (1.562-4.897)
Performed test	Yes	1.991 (0.24)	63.855	1	**0.000**	7.321 (4.49-11.93)
Knowing Symptoms	Yes	0.747 (0.26)	8.141	1	**0.004**	2.11 (1.264-3.526)
	Constant	2.691 (0.71)	14.314	1	0.000	14.753

Table 5 shows the sources of information about vitamin D among the study participants. The internet was the primary source used by the majority of participants (86.5% among Egyptian versus 82.4% among Jordanians), followed by university/school curricula (82.2% among Egyptian versus 66.4% among Jordanians) and health care providers (HCPs) (77.6% among Egyptian versus 62.7% among Jordanians). Differences in the use of sources were noticed between Egyptian and Jordanians. For example, Jordanians used TV channels, friends/relatives, and newspapers less than Egyptians to obtain information related to vitamin D (Table 5).

**Table 5:** Sources of information regarding vitamin D importance.

Source	Egyptians	Jordanians
Health care providers	77.6%	62.7%
Internet	86.5%	82.4%
News papers	63.2%	37.2
Friends/family member	72.1%	49.8%
TV channels	58.4%	26.5%
School/university Curricula	82.2%	66.4%

Table 6 shows the practices followed by participants to improve their vitamin D levels. These practices include sun exposure (84.6%), consumption of food rich in vitamin D (79.6%), monitoring blood vitamin D levels (49.5%), and using vitamin D supplements (36.7%). Some practices were prevalent among Jordanians compared to Egyptians, including monitoring blood levels of vitamin D and using vitamin D supplements (Table 6).

**Table 6:** Practices that are used by the study participants to improve vitamin D levels.

Practice	Egyptians	Jordanians	Total
Exposure to sun light	85.8%	83.5%	84.6%
Taking vitamin D supplements	27.2%	45.4%	36.7%
Consumption of foods rich in vitamin D	85.9%	73.8%	79.6%
Monitoring vitamin D levels	27.8%	69.3%	49.5%

## Discussion

The current study investigated the prevalence of vitamin D supplementation and associated factors. The results showed that about approximately one-third of the sample (36.7%) were using vitamin D supplements. Factors associated with vitamin D use included female gender, higher income, having a chronic disease, having a vitamin D blood level test, and awareness of deficiency symptoms.

Vitamin D is a nutrient that plays a vital role in calcium balance in the body. Calcium is essential for the bodily functions, including health bones and teeth and muscles contraction. A recent study indicates a strong correlation between serum vitamin D status and skeletal bone density among menopausal individuals [25]. Vitamin D deficiency has been linked to several health conditions in adults, such as muscle weakness, an increased risk of fractures, and cardiovascular/autoimmune diseases.

In the Middle East, vitamin D insufficiency and deficiency are common. A study examining vitamin D levels in adults in Jordan found that over 85% had vitamin D deficiency and insufficiency [10]. Among children in Jordan, approximately 40% had vitamin D deficiency, and about one-third had insufficiency [11]. In Egypt, a study conducted on children showed that about 70% of female children suffered from vitamin D deficiency or an insufficiency [13]. Another study conducted on adults reported that most participants suffered from vitamin D deficient/insufficient levels [12]. In Cairo, Egypt, the prevalence of vitamin D deficiency/insufficiency was reported to be 90% in the city [26]. Similar high rates of vitamin D insufficiency/deficiency were also reported in countries from the region, including Saudi Arabia, United Arab Emirates, Lebanon, Iraq, and Tunisia [14–18].

In the current study, more than a third of the sample reported using vitamin D supplements. In Jordan, 37–55% of participants reported using vitamin D supplement during COVID-19 pandemic [27–29]. In Benha, Egypt, approximately 18% of participants reported using vitamin D supplements [30]. In the UAE, vitamin D supplementation was reported to by 21.8% of children [31]. In Lebanon, vitamin D use ranged from 35.5% to 41% [32]. In Saudi Arabia, the prevalence of vitamin D use was reported to be high among medical students in Saudi Arabia [33]. During the pandemic in Saudi Arabia, the usage of vitamin D supplements reached 22% [34]. Thus, vitamin D supplementation is also common in the region.

In the current study, vitamin D use was associated with female gender, higher income, chronic diseases, laboratory vitamin D testing, and awareness of deficiency symptoms. In addition, slight differences in associated factors were observed between Jordan and Egypt. For example, age was associated with the use of vitamin D supplements among Egyptians, while educational level and awareness of symptoms were associated with the use of vitamin D supplements among Jordanians. Due to the limited studies conducted on the factors associated with the use of vitamin D supplements, the discussion will be expanded to include factors associated with the use of vitamins and dietary supplements in general. In agreement with the present study, a Canadian reported that women, older age, higher education, higher income, and chronic diseases were independent factors associated with vitamin use among adults [35]. In a study of adult workers in Tanzania, vitamin use was found to be associated with female gender [36]. In Asir, Saudi Arabia, dietary supplement use was found to be associated with old/middle-aged age, educational level, and female gender [37–39]. In a polish study, gender and chronic diseases were associated with dietary supplement use [40]. Among Koreans, education level and female gender were found to be associated with a higher likelihood of vitamin supplement use [41]. Pooled analyses of randomized controlled trials indicate that vitamin D supplementation enhances the healing of diabetic foot ulcers, suggesting its promise as an adjunctive therapy for chronic diseases [42]. Therefore, predictors of dietary supplement use vary slightly among different populations, including Egyptians and Jordanians.

Among Egyptians and Jordanians, laboratory testing for vitamin D was found to be associated with vitamin D supplement use. Similarly, awareness of vitamin D deficiency symptoms among Jordanians was associated with supplement use. Taking vitamin D supplements based on laboratory blood level tests and deficiency symptoms is crucial to avoid the side effects of vitamin D overdose. It has been reporting that vitamin D overdose is associated with serious complications such as hypercalcemia, hypercalciuria, and calcium deposition in soft tissues, and kidney stone formation [43–45]. Therefore, it is important to have clinical monitoring while taking Vitamin D supplements.

The study findings showed that the internet was the primary source of information about vitamin D for the majority of participants, followed by university/school curricula, and HCPs. Moderate knowledge of vitamin D was reported in a study encompassing 4 countries from the region [46]. The internet was the main source of information regarding vitamin D use in Egypt, while the internet and HCPs were the main sources in Jordan [46]. In Japan, the internet was reported to be a major source of information about dietary supplements [47]. Similar finding was found in Lebanon, where the internet was the main source of information used by athletes about dietary supplements [48]. It has been noted that HCPs and social media are sources of information about dietary supplements in Nigeria, Poland, and UAE [49–51]. Thus, it appears that the internet and HCPs are utilized globally as sources of information about dietary supplements.

The study showed that participants followed several practices to boost their vitamin D levels. These practices included sun exposure, consuming vitamin D-rich foods, monitoring blood vitamin D levels, and using supplements. A recent study from Jordan indicated a good level of knowledge and awareness regarding vitamin D sources [52]. In Egypt, it has been shown that insufficient sunlight exposure, a low-calcium diet, and contribute to vitamin D deficiency [26]. Thus, interventions should focus on promoting practices that can boost vitamin D levels in the body.

One limitation of the current study is its cross-sectional design, which shows associations not directionality. In addition, the study is self-reported and may therefore be affected by social desire bias and misremembering issues. Qualitative and longitudinal designs could be used in future studies to validate the current findings.

## Conclusion

Vitamin D supplementation is widespread across Egypt and Jordan, driven primarily by female gender, higher income, chronic conditions, prior laboratory testing, and awareness of deficiency symptoms. However, distinct regional variations exist; age influences supplement use in Egypt, whereas educational attainment and symptom awareness are key drivers in Jordan. Healthcare providers should proactively screen high-risk groups during routine check-ups while educating them on vitamin D health. Finally, mixed-methods studies are needed to explore the specific cultural and socioeconomic barriers that prevent equitable access to vitamin D testing and supplements in the region.

## Supporting information

S1 Appendix Excel file containing the raw data used for the analyses in this study. (XLSX)

S1 Checklist Global research questionnaire. (DOCX)
